# Complete Genome Sequences of Streptomyces albus Strain INA 01303

**DOI:** 10.1128/mra.00991-22

**Published:** 2022-11-16

**Authors:** D. V. Poshvina, T. A. Efimenko, M. V. Demiankova, A. A. Glukhova, T. D. Ivankova, O. V. Efremenkova, V. S. Sadykova, A. S. Balkin, A. S. Vasilchenko

**Affiliations:** a Laboratory of Antimicrobial Resistance, Institute of Environmental and Agricultural Biology (X-BIO), Tyumen State University, Tyumen, Russia; b Gause Institute of New Antibiotics, Moscow, Russia; c Laboratory of Biomedical Technologies, Institute for Cellular and Intracellular Symbiosis of the Ural Branch of the Russian Academy of Sciences, Orenburg, Russia; Indiana University, Bloomington

## Abstract

Here, we report the complete genome sequence of Streptomyces albus strain INA 01303, which was isolated from the Salt Lake Tambukan (Russia). The genome consists of a linear 6,840,896-nucleotide chromosome. This strain is predicted to produce a range of novel secondary metabolites with antibiotic activity.

## ANNOUNCEMENT

The genus *Streptomyces*, a unique subgroup of actinomycete bacteria, is best known for its ability to produce amounts of bioactive secondary metabolites with antimicrobial, antiparasitic, antitumoral, and immunosuppressant activity (1, 2).

Streptomyces albus strain INA 01303 was isolated from the littoral zone of the bottom sediments of Lake Tambukan (Stavropol Krai, Russia). The strain was isolated using the standard method of sowing soil suspensions on dense nutrient medium organic agar number 2 Gause (glucose, 1.0 g/L; peptone, 0.5 g/L; tryptone, 0.3 g/L; NaCl, 0.5 g/L; agar, 2.0 g/L; tap water, рН 7.2 to 7.4) (3). To prepare soil suspensions, 100 mg of soil was placed in 10 mL of sterile water and shaken for 10 min, then the suspension was diluted in ratios of 1:1,000 and 1:10,000 and sown on the medium in petri dishes. The culture was incubated at a temperature of 28°C for 14 days. The overnight culture of *S. albus* strain INA 01303, which was grown on the liquid number 2 Gause medium, was transferred for DNA isolation.

Genomic DNA was isolated by the phenol-chloroform method. Briefly, 350 μL of Tris-salt buffer (100 mM/L Tris-HCl, 20 mM/L EDTA, and 750 mM/L NaCl; pH 8.0) and 50 μL of lysozyme solution (10 mg/mL) was added to 50 μL of sediment of the studied culture and incubated for 30 min at 37°C. After that step, a 10% SDS solution was added to the mixture to a final concentration of 1%, 4.0 μL of a proteinase K solution (10 mg/mL), and 1.0 μL RNase I (10 mg/mL) and incubated for 30 min at 60°C. After extraction with a mixture of phenol-chloroform-isoamyl alcohol (25:24:1, vol/vol/vol) and subsequent extraction with chloroform-isoamyl alcohol (24:1, vol/vol), DNA was precipitated from the aqueous phase in 3 volumes of absolute ethanol with the addition of 10 M ammonium acetate (1:10, vol/vol) at −20°C overnight. After centrifugation and double washing with 70% ethanol, the DNA was dried and dissolved in 30 μL of deionized water. DNA concentration was estimated using Qubit 4.0 fluorimeter (Thermo Fisher Scientific, Germany). DNA quality was evaluated using a Nanodrop 8000 spectrophotometer (Thermo Fisher Scientific). Nanopore sequencing libraries were generated using the Native barcoding kit SQK-NBD112.24 (Oxford Nanopore Technologies, UK) protocol and sequenced on a SpotON flow cell vR9 (catalog number FLO-MIN106) using a MinION Mk1 device for 48 h in the Center of Shared Scientific Equipment “Microorganisms Persistence” of the Institute of Cellular and Intracellular Symbiosis of the Ural Branch of the Russian Academy of Sciences. The obtained Nanopore long reads were trimmed for minimal length (1,000 bp) and quality (q = 8) with Nanofilt (4). The total number of raw reads was 597,783 (957 Mb) with an *N*_50_ value of 6,234 bp. Reads were basecalled using Guppy basecaller v5.0.13 in «fast» mode.

The *de novo* genome assembly of the isolate was generated by Nanopore sequencing data using Flye v2.8.3 (5), and the assembly was polished with two rounds of Racon v1.4.12 (6). The resulting assembly contigs were analyzed using the QUAST software v5.0.2 (7) to assess assembly quality, as well as using BUSCO v4.1.1 (8) to evaluate assembly completeness. Genome annotation was performed locally using Prokka v1.14.6 (9). The prediction of the specialized metabolites biosynthetic gene clusters (BGCs) was performed with antiSMASH 6.0 using strict detection (10). All mentioned software has been used with default parameters unless otherwise specified.

The genome of Streptomyces albus strain INA 01303 consists of 6,840,896 bp with 85-fold overall coverage and average GC content of 73%. In the genome of strain INA 01303, a total of 7,893 protein-coding genes were annotated. The genome annotation contained 85 tRNAs, 21 rRNAs (5S, 16S, and 23S), 71 miscellaneous RNAs (miscRNAs), and 1 transfer-messenger RNA (tmRNA). Genome annotation revealed a number of biosynthetic gene clusters (BGCs). In total, 17 regions were predicted, covering 4 polyketide synthase (PKS), 3 nonribosomal peptide synthetase (NRPS), 4 PKS/NRPS, 4 terpene, 1 siderophore, and 1 ectoine biosynthetic gene clusters.

### Data availability.

The assembled genome sequence of Streptomyces albus strain INA 01303 was deposited in GenBank under accession number CP102119.1. Nanopore raw reads have been deposited in the NCBI Sequence Read Archive under accession number SRR20718915 (BioProject number PRJNA863464 and BioSample number SAMN30035760).
